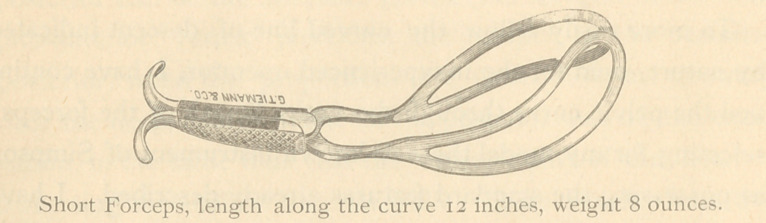# The Continued Pelvic Curve in the Obstetric Forceps, Etc.

**Published:** 1885-07

**Authors:** Edward Warren Sawyer

**Affiliations:** Chicago


### THE CONTINUED PELVIC CURVE IN THE OBSTETRIC FORCEPS, WITH REMARKS ON FORCEPS IN GENERAL.

*By* EDWARD WARREN SAWYER, A. M., M. D., *Chicago.*

[Read before the Chicago Medical Society, June 15th, 1885.]

The obstetric forceps is defined by Velpeau and by writers generally, as a variety of pincers intended to draw the fœtus from the mother. But the same definition applies to two distinct classes of extracting instruments whose offices are diametrically opposed to each other. The one, of which the cephalotribe may be taken as the representative, is devised for the sole purpose of destroying the contour of the head, and, as a result, the destruction of the fœtus. The office of the forceps, on the other hand, is to extract a living fœtus without injury. I may go a step further and say that the perfect obstetric forceps is an instrument with which it is practically impossible to injure the mother or fœtus.

The object of this communication is to call attention to several features which this instrument must possess to be the harmless extractor. Extensive series of measurements have been made, and repeated by numerous authorities, of the constant unyielding canal through which the fœtus is to pass, and of the constant unyielding head of the fœtus which is to be passed. From these observations certain constant dimensions are established, which are known as standard, for the bony canal and the head of the fœtus.

If we turn for a moment to a more particular examination of the forces invoked in the use of the forceps, it will be seen that they are practically two: Compression of the head, by which its dimensions are lessened within safe limits, and traction, by which the expulsive forces of nature are reinforced. Both these forces are conjointly invoked whenever this instrument is used.

It is the first-named force, that of compression of the head, which contains the element of danger to the offspring, and consequently that which should be under the complete control of the operator. Repeated observation has shown that the various transverse diameters of the fœtal head may be lessened by compression a trifle more than one-half inch, without danger to the fœtus. In fact, it is probable that in the great majority of labors, compression takes place to this degree, caused by the natural forces alone.

Given then the standard or usual size of the bony pelvis, the fœtal head and the safe limit of compression, it becomes an easy task to construct an instrument, that portion of which that is to grasp the head within the pelvis, shall possess certain dimensions and be called standard. That such dimensions should be described, seems warranted from the foregoing; and yet I am not aware that any text-book contains such a description of standard forceps for the guidance of a student who is to select an instrument.

In may be stated in general terms that the various diameters of the flexed fœtal head which would be grasped by the forceps in vertex labors, are three and three-fourths inches. Subtracting the margin of one-half inch for safe compression, there remains a space of three and one-fourth inches which should exist between the compression surfaces of the blades at the moment of greatest compression, or when the inner surfaces of the handles are in contact.

The instruments which I now exhibit have respectively two and one-fourth inches, Tannier, and two and one-fourth inches, Hodge. They are, therefore, not forceps, according to our definition, but crushing instruments; and while their compressing force may be to a certain extent limited and controlled by the skillful hand, it is always dangerous in the hands of the ordinary practitioner.

This feature I regard second in importance to none other. It being accepted, the long diameter of the ellipse bounded by the conjoined grasping curves of the blades, should next be considered. Experience has shown that this ellipse should be five and one-half inches in length. It is open at both extremities. When the handles are closed the distal opening of the ellipse, corresponding to the separation of the tips of the blades, may be three-fourths of an inch. While the anterior opening corresponding to the distance between the shanks of the blade, at five and one-half inches from the tip, should be two and one-half inches. Should this distance be materially lessened, injury to the head cannot be avoided when compression is made. In these instruments already shown, which are not unlike many in this respect to be found in the shops, there is a separation of but one-half an inch at the tip, and an opening of one and one-fourth inches at the anterior opening of the ellipse in Tannier, and in reality no opening at all in the instrument of Hodge.

The width of the blade at the widest part may be one and one-half inches; but this is a point of less importance than that next in order. In selecting an instrument a straight edged ruler should be placed at right angles with the long diameter of the blade upon its inner surface, to determine if the outer edge of the blade and the border of the fenestra are on the same plane. If they are not the instrument should be rejected on this ground if upon no other; for upon this disparity depends in a great measure the injury inflicted to the fœtal scalp.

The next important feature of the forceps is the lock. All devices for locking the blades belong to two classes. Both admit of almost unlimited lateral hinge movement, but one variety only, the mortise lock of Smellie, admits of a certain additional movement, backward and forward, by means of which the end of the handles can be slightly slipped past each other. The second variety of lock is a button, or thumb-screw which so fixes the blades as to preclude any degree of backward and forward movement of the handles. With this explanation these locks may be called respectively loose and fixed.

A simple inspection will enable one to determine which lock is safe, simple in construction and easy of operation, while the other is dangerous to the child, complicated and usually difficult of operation. The infinite superiority of the loose mortise is at once accepted in recalling a single fact in practice: It is only in the exceptional case that the blades grasp the head exactly upon its sides, or upon any two opposite and parallel planes. In the majority of cases the head plane occupied by one blade is oblique with reference to the other. It is obvious then that if no mobility exists in the lock that the blades press unevenly upon the head; indeed only one border of the blade may rest upon the head, and receive the entire compressing force which should be shared by both borders equally. As a result I have seen one edge sink to a shocking depth into the skull. Even a slight degree of mobility at the lock is considerably increased at the ends of the blades.

Before leaving this part of the instrument I desire to call attention to a special feature which, for want of a better name, I have called the heel of the blade. This rounded prominence upon the lower border of the blade, nearly opposite the beginning of the fenestra, finds its most dangerous exaggeration in the instrument known as the Davis forceps, although many instruments perpetuate what I feel justified in pronouncing a dangerous fault. It increases the weight and bulky appearance of the instrument. It does not add to the grasping power of the blades; for the head is usually grasped at a point beyond this prominence. Above all it is a real menace to the woman's soft-parts, which it too often succeeds in tearing. In fact, in a given case when this instrument was being used by a highly competent practioner, I had an opportunity of an ocular demonstration of fact that the margin of the woman's vulva was torn by these heels before the head itself had reached the perineum.

It seems proper in this connection to call attention to another fact, viz.; that in compressing the fœtal head the closest imitation of nature is the safest to follow; and that a moment of compression should be at once followed by a loosening of the head. Consequently those instruments which admit of the handles being fixed together, by means of a screw or other device, can not be included among safe extracting instruments.

The remaining part of the forceps is called the handle, of which the length, largeness, shape and other particulars are as varied as the ingenuity of the inventors. In fact it is only in this part of the instrument that any inventor can claim any pronounced originality since the days of Smellie, who added the pelvic curve, and the mortise lock already alluded to.

In one particular, however, the handle should be considered. It is obvious that the greater the leverage possessed by the handles, the more easily is the compressing power of the instrument to be invoked. Consequently the length of the handles should be such as to allow the operator to reserve his forces for difficult traction.

The rounded space just in front of the lock, allowed by the separation of the shanks, is a feature accredited to Ramsbotham, the elder. It admits of the introduction of the finger, or a napkin. It has special value, as a point of traction when the greatest degree of compression is not demanded. Besides this traction point Simpson has added the shoulders at the beginning of the handles, and others have copied from him in a ring to receive the finger, with some disadvantage.

The blades of an instrument to be entirely reliable should be constructed of the best of steel, which should be at least one-fourth of an inch square at the shank ; from this size the material may be thinned to 3—32 of an inch in thickness at the tip of the blades. Slighter material than this I have seen so yielding as to allow the blades to be pulled off the head.

The earliest cases of forceps labors, which occured under my observation, demonstrated to me one fact which further experience has strengthened and confirmed. In passing through the pelvic excavation, the part of the head in the posterior half of the woman's pelvis, (the sinciput in the occipito-anterior vertex positions, the occiput, in the occipito-posterior) when the proportions are usual, is forced to sweep over the deep, curved plane of the posterior wall of the excavation, in the same time that the anterior part of the head is traversing the shallow anterior wall. There results from this inequality of distances traversed, a movement of extension of the fœtal head in occipito-anterior positions, and the opposite movement, or flexion, in occipito-posterior positions.

If an index be fixed to the head, as for example the handles of a forceps, in such a manner as to follow the directions given to the head by the unaided efforts of nature, an irregular curved line would be described forward and upward which would have its termination, at the moment of the escape of the head, at a point near the umbilicus of the woman.

Throughout the length of the bony portion of the parturient canal, this curved line should be the guiding line to the attendant who attempts to reinforce the expulsive force of the uterus with the forceps. The curved direction which the handles will take is in an exact relation to the degree of descent of the head, and is to be learned practically at the bedside.

Of course it is not a question here of those rare cases where the forceps is applied when the head is yet unengaged above the superior strait.

When the head has almost freed the bony section of the canal, and is retained chiefly by the soft parts, the writer has for a long time in practice interfered with the natural movement of evolution executed by the head (extension or flexion) and has compelled the head to escape in a decidedly flexed state, thus presenting its smallest diameter to the ostium vaginæ. This interference he holds to be the greatest safe guard of the perineum.

To more easily follow the curved line of descent indicated by nature, even for the inexperienced operator, I have continued the pelvic curve through the entire length of the forceps; selecting for my model the well-known instrument of Simpson as possessing the standard features already described. I have also added an entirely new handle which has been found efficient.

Continued pelvic curve in profile.

One advantage remains to be mentioned: In those cases where the occiput presents posterior, and it is decided to deliver in this position, the curve of this instrument enables the operator to grasp the head well forward over the ears. Under these circumstances this is the only instrument which in my hands has not slipped over the occipital pole.

*Long Forceps, length along the curve 17 inches, weight 23 ounces.

The smaller instrument has all the essential features of the larger except force. It is only suitable, obviously, to very low head positions; but in my own practice and in that of others of my acquaintance its use is invoked many times oftener than that of the long instrument.

Short Forceps, length along the curve 12 inches, weight 8 ounces.

A feature which merits notice is the baking of the hard rubber handles by the Tiemann process which greatly lessens the danger of the handles being carriers of filth and infection.

---

*First mentioned in the Chicago Journal and Examiner, May 1878, page 499. Note.—Exhibited to the American Gynæcological Society, Chicago, Sept., 1884.

## Figures and Tables

**Figure f1:**
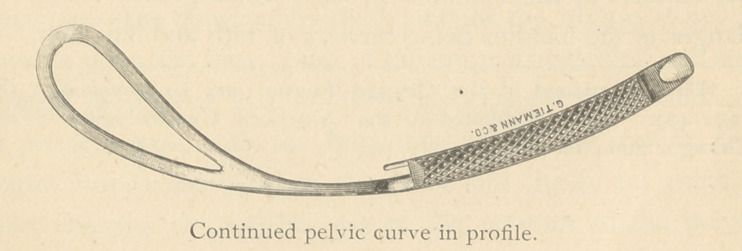


**Figure f2:**
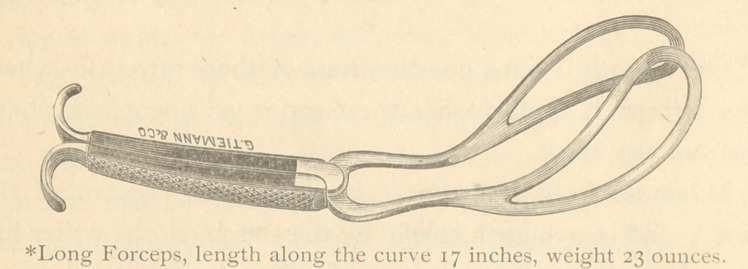


**Figure f3:**